# Case report: Revascularization failure in NF1-related moyamoya syndrome after selumetinib: A possible pathophysiological correlation?

**DOI:** 10.3389/fped.2023.1051026

**Published:** 2023-02-27

**Authors:** Cristina Chelleri, Marcello Scala, Patrizia De Marco, Monica Traverso, Marzia Ognibene, Irene Bruno, Gianluca Piccolo, Pasquale Striano, Mariasavina Severino, Federico Zara, Maria Cristina Diana, Marco Pavanello

**Affiliations:** ^1^ Pediatric Neurology and Neuromuscular Disorders Unit, IRCCS Istituto Giannina Gaslini, Genoa, Italy; ^2^ Department of Neurosciences, Rehabilitation, Ophthalmology, Genetics, Maternal and Child Health (DINOGMI), University of Genoa, Genoa, Italy; ^3^ Medical Genetics Unit, IRCCS Instituto Giannina Gaslini, Genoa, Italy; ^4^ Department of Pediatrics, Institute for Maternal and Child Health Burlo Garofolo, Trieste, Italy; ^5^ Neuroradiology Unit, IRCCS Istituto Giannina Gaslini, Genoa, Italy; ^6^ Department of Neurosurgery, IRCCS Istituto Giannina Gaslini, Genoa, Italy

**Keywords:** neurofibromatosis type, selumetinib, moyamoya vasculopathy, brain MRI, VEGF

## Abstract

Neurofibromatosis type 1 (NF1) is a neurocutaneous syndrome caused by pathogenic variants in the *NF1* gene, encoding a multidomain inhibitor of Ras activity. Thus, NF1 is considered a RASopathy and drugs targeting the RAS/mitogen-activated protein kinase (MAPK) pathway, such as the MAP kinase (MEK) 1/2 inhibitor Selumetinib, are promising therapeutic options to treat NF1-associated tumors, especially plexiform neurofibromas and optic way gliomas. However, surgical treatment is often required for NF1-related cerebrovascular manifestations, such as moyamoya syndrome (MMS). We report a case of an 8-year-old patient receiving Selumetinib at the dose of 25 mg/m2 orally 2 times a day as a treatment for many plexiform neurofibromas. He suffered from two close strokes and brain MRI revealed a severe cerebral vasculopathy consistent with MMS, with marked stenosis of both the internal carotid arteries. A two-step surgical revascularization procedure was performed, consisting of a direct by-pass with an encephalo-mio-synangiosis (EMS) followed by encephalo-duro-arterio-synangiosis (EDAS). Surprisingly, despite the surgical technical success, follow-up MRI revealed lack of the expected revascularization. Selumetinib is a powerful therapeutic option in the treatment of severe NF1-related tumors. However, our findings suggest that this drug may interfere with cerebral neovascularization in patients with MMS requiring surgical revascularization. This is supported by the crucial role of the Vascular-Endothelial Growth Factor (VEGF), whose signaling pathway involve MAPK, as promoter of the neovascularization. Our observations suggest to adopt an imaging surveillance strategy to prevent unfavorable surgical outcome in patients with NF1-associated MMS receiving Selumetinib, and that priority should be given to surgical revascularization.

## Introduction

Neurofibromatosis type 1 (NF1) is a clinical heterogeneous neurocutaneous syndrome caused by pathogenic variants in the *NF1* gene (1). The product of this gene, neurofibromin, is a multidomain inhibitor of Ras activity, which is crucial for the regulation of intracellular signal transduction for cell growth and differentiation (2). Therefore, NF1 is considered a member of the RASopathies, a group of genetic conditions caused by mutations in genes encoding for members of the RAS/mitogen-activated protein kinase (MAPK) pathway (3).

RASopathies are characterized by variable association of dysmorphism, developmental delay, congenital multiorgan abnormalities, and increased risk of cerebrovascular disorders and cancer (3). In particular, optic pathway gliomas (OPGs) and plexiform neurofibromas (PNs) are especially common in NF1 patients, as well as a form of cerebral vasculopathy characterized by progressive occlusion of distal intracranial carotid arteries and their proximal branches, known as moyamoya syndrome (MMS) (4). This vasculopathy may lead to severe ischemic neurological sequelae if left untreated, and often requires surgical revascularization procedures (5).

In the last few years, drugs interfering with the MAPK pathway have been proposed and used in the treatment of NF1-related tumors, especially OPG and PNs (6). Selumetinib, a MAP kinase (MEK) 1/2 inhibitor, has proven effective in the achievement of durable tumor shrinkage in inoperable PNs and prolonged disease stability in patients with recurrent or progressive OPGs (7–9). The safety profile of this drug is good and most common side effects only include skin toxicity (∼65%), diarrhea (∼60%) and increased creatine kinase levels (∼60%) (10). However, despite these really promising results, the need for further larger-scale randomized controlled studies exploring the long-term outcome and the side effects has been suggested (10).

We report a case of a NF1 patient receiving Selumetinib for plexiform neurofibromas and failing to develop neovascularization after revascularization surgery for concomitant symptomatic MMS.

## Case description

**Figure 1:**
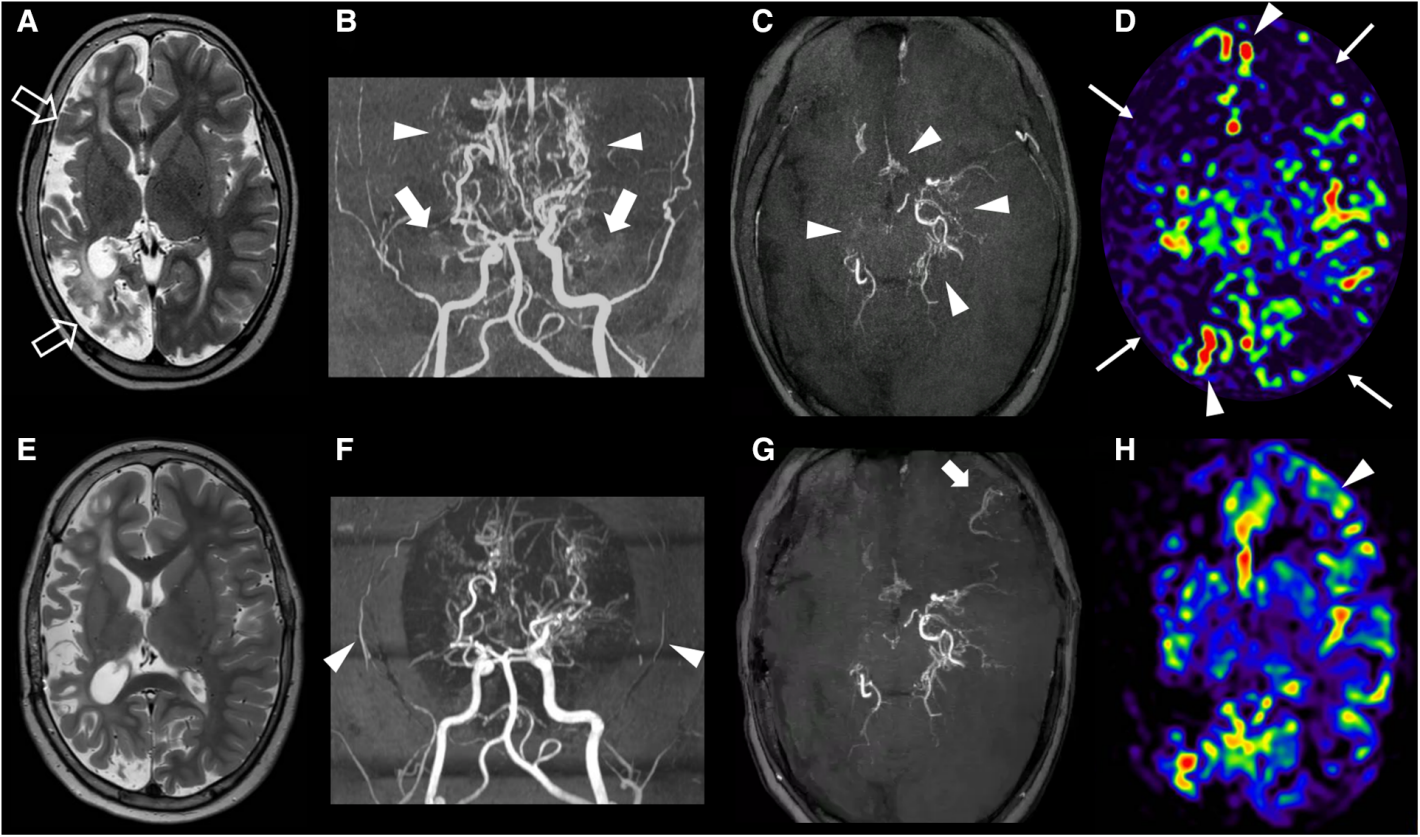
Clinical and imaging findings. Brain MRI and MR angiography of the subject performed before revascularization at 8 years of age (**A–D**) and at last follow-up, after eleven months (**E–H**). A) Axial T2-weighted image shows a chronic arterial ischemic infarct in the right cerebral hemisphere (empty arrows) with relative sparing of the mesial frontal and insular regions. (**B,C**) Brain MR angiography with 3D reconstruction, frontal view (**B**) and axial MinIP reconstruction (**C**) demonstrate bilateral stenosis of the right supraclinoid portions of the internal carotid arteries with absent flow signal in the middle cerebral arteries (thick arrows) and anterior cerebral arteries, and multiple moyamoya collaterals (arrowheads). Note the initial distal stenosis of the left posterior cerebral artery. (**D**) Brain arterial spin labeling MR perfusion reveals reduced signal in the right cerebral hemisphere and in the left anterior frontal and parietal lobes (thin arrows) with bilateral arterial transit artifacts (arrowheads). (**E**) Follow-up axial T2-weighted image performed 11 months after the revascularization procedure shows no other ischemic infarcts. (**F,G**) Corresponding MR angiography images reveal reduction of flow signal in the superficial temporal arteries (arrowheads), absence of collateral formation at surgical site on the right, and a limited region of revascularization in the left fronto-basal region (thick arrow). (**H**) Arterial spin labeling MR perfusion demonstrated increased CBF only in the left fronto-basal region (arrowhead).

An 8-year-old boy presented to our NF1 referral center after the recent identification of cerebral vasculopathy on brain MRI. He was diagnosed with NF1 at the age of 2 years for the presence of Cafè-au-lait macules and genetic testing confirmed the diagnosis through the detection of the *de novo* NF1 (NM_000267.3): c.6179T > G (*p*.Leu2060*) pathogenic variant in the *NF1*. After the identification of multiple inoperable plexiform neurofibromas of the spine, treatment with Selumetinib was started at the dose of 25 mg/m^2^ orally 2 times a day when the patient was 4 years old. His clinical history was also remarkable for bilateral renal artery stenosis at the age of 5 years treated with angioplasty and for two episodes of stroke at the age of 2 and 6 years. The second episode occurred despite the prophylactic aspirin therapy at the dose of 100 mg orally per day started in combination with levetiracetam at the dosage of 500 mg orally 2 times a day after the first stroke. Brain MRI at 8 years of age showed a large chronic infarct in the right middle and posterior cerebral arteries territories (Figure 1A) while MR angiography demonstrated marked stenosis of both supra-clinoid portions of the internal carotid arteries, with absent flow signal in the middle cerebral arteries and anterior cerebral arteries and extensive moyamoya collaterals (Figures 1B,C). Initial stenoses were present also at the level of the posterior cerebral arteries. Brain perfusion MR studies revealed areas of reduced perfusion in both the right and left hemispheres (Figure 1D). Globally, these findings were suggestive of a severe form of MMS.

Since the cerebral vasculopathy was symptomatic, surgical revascularization was indicated. Since the suspension of Selumetinib is not currently indicated in the drug brochure and the patient was severely symptomatic, we decided not to stop the Selumetinib administration during and after surgery. He was first treated with a left encephalo-mio-synangiosis (EMS). A month later, a right encephalo-duro-arterio-synangiosis (EDAS) was also performed. However, follow-up brain MRI studies performed 4 and 11 months after the procedures, respectively, showed an extremely limited neovascularization, only involving a small portion of the left frontal basal cortex (Figures 1E–H).

## Discussion

The MAPK pathway is an extremely important intracellular signaling pathway involved in cell proliferation and survival, and its constitutive activation is implicated in the pathogenesis of a variety of malignancies (11). Therefore, intense research has been conducted on this pathway for the development of pharmacologic inhibitors potentially useful in cancer (12). Following ligand binding from an array of growth factors receptors, there is an activation of the RAS–RAF pathway which eventually converges into the activation of MEK1 and MEK2 (13). These are dual-specificity kinases catalyzing activating phosphorylation events in ERK1 and ERK2, which in turn catalyze the phosphorylation of several intracellular substrates modulating mitosis, cell differentiation and motility, metabolism, apoptosis, and angiogenesis (13, 14). Therefore, MEK 1/2 are therapeutically targetable bottlenecks in the MAPK pathway, especially towards the inhibition of processes involved in tumorigenesis (13).

MEK inhibitors bind to and inhibit MEK, leading to the inhibition of MEK-dependent cell signaling (15). Therefore, these drugs counteract among other processes the abnormal cell proliferation and programmed cell death involved in tumorigenesis (15). MEK inhibitors have been employed in several types of human cancers and their use has been recently proposed in the treatment of NF1-related tumors (15). In particular, Selumetinib has proven effective in reducing tumor mass or slowing tumor growth in a high percentage of patients with inoperable PNs and recurrent and/or progressive OPGs, also alleviate correlated symptoms such as excruciating pain (7–9). Furthermore, their satisfactory safety profile has favored the implementation of their use in clinical practice (10).

NF1 is a complex and heterogeneous medical condition, with limited correlations between the individual genetic background and the disease manifestations (4). As such, each patient may show a distinctive combination of clinical features, including those mentioned in the diagnostic criteria (4, 16). Cerebral vasculopathy have been reported in 3%–7% of pediatric NF1 patients, manifesting as elongation/tortuosity of cerebral vessels, arterial stenosis, aneurysms and MMS (17, 18). The latter is a significant cause of pediatric stroke and often requires surgical treatment based on clinical and neuroradiological evaluation (5). Surgical revascularization procedures, including direct or indirect techniques, aim at the development of a neovascularization to overcome the limited blood flow caused by artery occlusion (5). Appropriate neoangiogenesis is crucial to achieve this goal and the Vascular Endothelial Growth Factor (VEGF) plays a pivotal role in this process (19). The binding of VEGF to its receptor leads to the activation of the PLC*γ* (phospholipases C *γ*) and PKC-Raf kinase-MEK-mitogen-activated protein kinase (MAPK) pathway, which initiates DNA synthesis to promote endothelial cell proliferation towards new blood vessel formation (13, 19).

In our patient, we could not observe the expected neoangiogenesis after two technically successful surgical revascularization procedures performed for symptomatic MMS. Given the extremely relevant role of VEGF in this process, these findings lead to speculate that the inhibition of the MAPK pathway operated by Selumetinib might have contributed to the lack of new vessels development in the reported subject. Although further reports and dedicated studies will be fundamental to possibly confirm this observation, this case suggests that the use of Selumetinib might limit the efficacy of surgical revascularization procedures in NF1 patients with moyamoya vasculopathy. As such, we suggest that a careful evaluation of the timing of surgical intervention would be advisable in patients that are candidates to Selumetinib treatment. This is particularly relevant since drug withdrawal may be difficult, especially when inoperable tumor masses can cause life-threatening complications (e.g., tracheal compression due to a large PN).

Although caution is necessary, the possible correlation between drug treatment and the surgical outcome in our patient might suggest to adopt a strategy to prevent unfavorable outcome in patients with NF1-associated moyamoya vasculopathy receiving Selumetinib. A reasonable step would be to withhold Selumetinib therapy until the signs of neovascularization are detectable. This could be assessed through a MRA performed 6 months after the revascularization procedure. Additionally, we suggest that the use of other imaging techniques may be helpful to monitor changes in the blood flow of the donor vessel. In this regard, a regularly performed transcranial color-coded duplex sonography (TCCS) (at 1 week, 1 month, 3 months, 6 months, and 1 year after surgery) is a safe and helpful imaging method to quantify the blood flow of donor, recipient, and bypass arteries, providing a relevant information to patient management (20).

In this study, we report a patient with severely symptomatic NF1-associated MMS receiving Selumetinib, in whom surgical revascilarization revealed unsuccessful in promoting cerebral neovascularization. Although further studies are necessary to confirm this association, our observations suggest a possible pathophysiological link between Selumetinib administration and deficient cerebral neovascularization in patients with NF1-associated moyamoya vasculopathy, based on the molecular mechanisms involved in the VEGF-MEK/ERK signaling pathway. We thus suggest that a cautious imaging strategy to help prevent unfavorable surgical outcome would be advisable in NF1 subjects with MMS undergoing surgery, and that priority should be given to revascularization surgery in these patients.

## Data Availability

The raw data supporting the conclusions of this article will be made available by the authors, without undue reservation.
